# Extended Target Recognition in Cognitive Radar Networks

**DOI:** 10.3390/s101110181

**Published:** 2010-11-11

**Authors:** Yimin Wei, Huadong Meng, Yimin Liu, Xiqin Wang

**Affiliations:** Department of Electronic Engineering, Tsinghua University, Beijing 100084, China; E-Mails: weiym@mails.tsinghua.edu.cn (Y.M.W.); yiminliu@tsinghua.edu.cn (Y.M.L.); wangxq_ee@tsinghua.edu.cn (X.Q.W.)

**Keywords:** cognitive radar network, radar waveform design, target recognition

## Abstract

We address the problem of adaptive waveform design for extended target recognition in cognitive radar networks. A closed-loop active target recognition radar system is extended to the case of a centralized cognitive radar network, in which a generalized likelihood ratio (GLR) based sequential hypothesis testing (SHT) framework is employed. Using Doppler velocities measured by multiple radars, the target aspect angle for each radar is calculated. The joint probability of each target hypothesis is then updated using observations from different radar line of sights (LOS). Based on these probabilities, a minimum correlation algorithm is proposed to adaptively design the transmit waveform for each radar in an amplitude fluctuation situation. Simulation results demonstrate performance improvements due to the cognitive radar network and adaptive waveform design. Our minimum correlation algorithm outperforms the eigen-waveform solution and other non-cognitive waveform design approaches.

## 1. Introduction

The importance of radar target identification is widely recognized and it has become one of the major concerns in radar surveillance and homeland security applications [1]. Normally, radar high range resolution profile (HRRP) is used as an important feature in radar automatic target recognition (ATR) [2–5], since it contains target structure signatures, such as target size, scatterer distribution, *etc.* [6]. In [7], a target impulse response was introduced to model target scattering behavior, and an optimal transmit waveform and receiver filter pair was proposed for extended target detection in additive Gaussian noise. By maximizing the output signal-to-noise ratio (SNR), Bell [7] derived an eigen-waveform solution under a total energy constraint. The eigen-waveform solution has been heuristically extended to tackle the multi-extended-target identification problems in [8], where the transmit waveform is designed to maximize the average (weighted average, generally) Euclidean distance or Mahalanobis distance (in additive colored noise) between different hypotheses.

Recently, Haykin proposed the novel idea of cognitive radar [9], one of whose most important characteristics is closed-loop operation. With the feedback structure from the receiver to the transmitter, waveforms can be adaptively optimized based on prior knowledge about targets and environments to improve system performance and efficiency. Many prior attempts have focused on target recognition using waveform adaptation in cognitive radar. Haykin [9] suggested that such a cognitive radar system can be represented using a Bayesian formulation whereby many different hypotheses are given a probabilistic rating. Based on this idea, Goodman [10] proposed the integration of waveform design techniques [8] with a sequential-hypothesis testing (SHT) framework [11] that controls when hard decisions may be made with adequate confidence [12]. He also compared two different waveform design techniques for use with active sensors operating in a target recognition application. One considered by Bell [7] is based on a maximization of the mutual information between a random target ensemble and the echo signal, and the other is based on eigenvectors of the weighted autocorrelation matrix proposed by Guerci [8] and Pillai [13]. To make full use of the transmit energy under a maximum modulus constraint, an adaptive single-tone waveform design algorithm was proposed in the same situation [14]. The target hypotheses were further extended to statistical characterization by power spectral densities in [15] where waveforms are matched to the target class rather than to individual target realizations.

However, several issues must be considered when applying impulse response to radar ATR. The most important of these is the well-known target-aspect sensitivity [6]. Since the impulse response represents the projection of the target scattering behavior onto the radar line of sight (LOS) [6], variation in the target aspect will lead to different impulse responses. Without a priori knowledge of the target aspect angle, 360-degree template matching is inevitable, which will cause significant degradation of recognition accuracy, especially in situations with a large number of hypotheses. In most surveillance applications, the target, such as an aircraft or ship, is moving and its major axis (heading direction) is approximately parallel to its velocity vector [16]. Therefore, the aspect angle can be acquired by estimating the target velocity via tracking. This approach works effectively when dealing with non-maneuvering (constant velocity and acceleration) targets. However, the ability to handle maneuvering targets is still lacking [17]. Another major issue is the amplitude sensitivity of returned echoes. This comes from the fact that the amplitude of a returned echo is affected by multiple factors such as target distance, antenna gain, receiver gain, and weather conditions [6]. Since some of these factors are unpredictable and unstable, the amplitude of a returned echo is usually unknown and variable. Therefore, the signal models [7,8,10] that assume the amplitude of returned echoes is deterministic and accurately known are not suitable for practical applications.

In this paper, we address the problem of extended target recognition in cognitive radar networks whose constitution was described by Haykin [18]. A cognitive radar network system should incorporate several radars working together in a cooperative manner with the goal of realizing a remote-sensing capability far in excess of what the radar components are capable of achieving individually [18]. In our extended target recognition application, the radar network can provide more robust detection performance [19,20], more accurate position estimation [21], and the most importantly, more reliable target aspect angle for each radar. Since the velocity of the target can be directly estimated using the Doppler frequencies measured by the individual radars, the issue of target-aspect sensitivity can be solved even for maneuvering targets. Also, because the radar stations are located across a large area, the sensor network is able to obtain returned echoes from multiple aspects at the same time, which leads to a significant improvement in the efficiency and robustness of the recognition.

The main contribution of this paper is to extend the above mentioned closed-loop active target recognition radar system [10] to the case of a centralized cognitive radar network. Once all radars have performed their observations, the target aspect angle for each radar is calculated. The joint probability of each target hypothesis is then updated using all the observations from different radars based on their aspect angles. The next transmit waveform for each radar is designed according to the joint probabilities of the target hypotheses. Such interrogation repeats until hard decisions can be made with adequate confidence. We also contribute by considering the amplitude uncertainty of the returned echoes. The ideal echo signal, which is the convolution of the transmit waveform with the target impulse response, is multiplied by a random complex coefficient in our signal model. A generalized likelihood ratio (GLR) based SHT framework in which the unknown parameters are replaced with their maximum likelihood estimates (MLE) is employed to update the joint probabilities of target hypotheses instead of the likelihood ratio based approach in [10]. Although the GLR test is not optimal, it appears to work quite well in practice. Finally, the adaptive waveform design algorithm described in [8,10] is applied to the cognitive radar network. Because the eigen-waveform solution [7,8,10] is no longer suitable for the amplitude fluctuation situation, a minimum correlation algorithm is proposed and compared with the algorithm based on average Euclidean distance.

In the next section, we define the problem and system model. The GLR based SHT framework and the centralized Bayesian update equations are presented in Section 3. In Section 4, the improved adaptive waveform design algorithm is detailed. Simulation results are shown in Section 5, and finally, Section 6 concludes the paper.

## 2. Problem Description and Modeling

We consider the target recognition problem in which one of *M* possible targets is known to be present. The position and velocity of the target are assumed to be known by the radars. Our objective is to identify the target accurately and quickly. In this section, we first describe the centralized cognitive radar network framework for solving the issue of target-aspect sensitivity. Then, a parametric measurement model is developed by considering the amplitude uncertainty of the echo.

### 2.1. System Model

In most radar surveillance applications, targets are far from the radar station and move horizontally, make it reasonable to assume that the targets and the radar station are located in the same horizontal plane [1–6]. A two-dimensional target model is used in our analysis.

**Figure 1.**
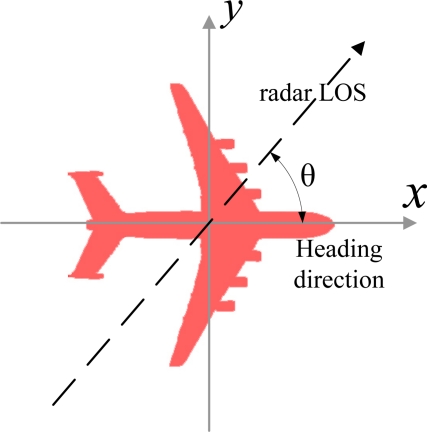
Definition of target aspect angle.

As shown in Figure 1, each target hypothesis *H_j_* is characterized by a set of impulse responses *g_θ,H_j__*(*t*) measured offline from every aspect angle *θ*. The aspect angle *θ* is defined as the angle between the major axis of the target (heading direction) and the radar LOS. For most of the targets, the major axis has approximately the same direction as its velocity vector [16]. The aspect angle can be acquired by estimating the target velocity vector and the position relative to the radar.

**Figure 2.**
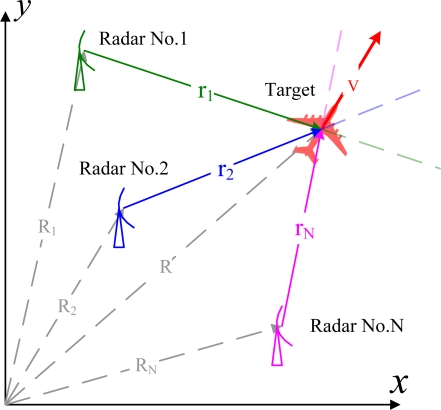
Top view of the cognitive radar network in an x-y coordinate system.

A cognitive radar network with *N* radars is introduced to solve the issue of target-aspect sensitivity. As shown in Figure 2, the radar stations are located in the same two-dimensional space in which the target moves. The position of the *i*th radar station is denoted as *R⃗_i_* = *R_ix_x̂* + *R_iy_ŷ*, where *x̂* and *ŷ* are the axis unit vectors and *R_ix_* and *R_iv_* are the axis weights. The position of the target is denoted as *R⃗*. The relative position of the target with respect to the *i*th radar is then given by *r⃗_i_* = *R⃗* − *R⃗_i_*. According to our definition, for a target with velocity vector *v⃗*, the aspect angle of the *i*th radar *θ_i_* is expressed as *θ_i_* = arg (*r⃗_i_*) − arg (*v⃗*), where the function arg() returns the angle of a vector in the x-y coordinate system. With the observations from multiple radars located in different places, the radar network can provide a more robust detection performance [19,20] and more accurate position estimation [21]. More importantly, the velocity of the target can be directly estimated based on the Doppler frequencies measured from different angles [21]. Therefore, the estimated aspect angle can be calculated by:

(1) $${\hat{\theta }}_{i}=\text{arg}\left({\hat{\vec{r}}}_{i}\right)-\text{arg}\left(\hat{\vec{v}}\right)$$

where $\hat{\vec{r}}$ and $\hat{\vec{v}}$ are the estimated relative position and velocity.

### 2.2. Measurement Model

Only backscattering is considered in our network, and interference among the radars is ignored. This makes the network easy to implement since each radar station can operate using a different frequency band. For the *i*th radar in the network, when *H_j_* is present, the echo signal *s_i_*(*t*) is determined by the aspect angle *θ_i_* and the transmitted waveform *u_i_*(*t*). It is given by:

(2) $$s_{i}\left(t\right)=a_{i}e^{j2\pi f_{\theta_{i}}t}\left[g_{\theta_{i},H_{j}}\left(t\right)*u_{i}\left(t\right)\right]+n_{i}\left(t\right)$$

where *g_θ_i_,H_j__*(*t*) is the target impulse response for the *j*th hypothesis at aspect angle *θ_i_*, * denotes the convolution operator, *f_θ_i__* is the target Doppler frequency at aspect angle *θ_i_*, *a_i_* is a random complex coefficient representing the amplitude and initial phase uncertainties of the echo signal, and *n_i_*(*t*) is additive white complex Gaussian noise at the receiver. The Doppler frequency *f_θ_i__* is given by *f_θ_i__* = −*f_c_*(2|*v⃗*|cos*θ_i_*/*c*), where *f_c_* is the carrier frequency and *c* is the speed of light.

Because most of the radar systems are digitized, and for the convenience of simulation, we use a discrete-time formulation to replace the model in Equation (2). The estimated Doppler speed $\hat{\vec{v}}$ and the estimated aspect angle *θ̂_i_* are used to eliminate the Doppler phase shift inside the echo signal by multiplying the inverse phase sequence with frequency ${\hat{f}}_{\theta_{i}}=f_{c}(2\left|\hat{\vec{v}}\right|\text{cos}{\hat{\theta }}_{i}/c)$. Although the estimated Doppler frequency *f̂_θ_i__* is different from the real value *f_θ_i__*, the slight difference can be ignored. After phase compensation, the echo signal is then given by:

(3) $$s_{i}=\alpha_{i}g_{\theta_{i},H_{j}}*u_{i}+n_{i}$$

where all the continuous-time signals are sampled using the same sampling interval *T_s_*, *α_i_* is the digitized random coefficient given by *α_i_* = *T_s_a_i_*, **u***_i_* is an *L_u_* × 1 complex vector representing the transmit waveform, **g***_θ_i_,H_j__* is an *L_g_* × 1 normalized complex vector representing the impulse response of *H_j_* at aspect angle *θ_i_*, **n***_i_* is an *L_s_* × 1 (*L_s_* = *L_u_* + *L_g_* − 1) complex vector representing the circularly symmetric zero-mean complex Gaussian noise with known variance $\sigma_{i}^{2}$, and **s***_i_* is an *L_s_* ×1 complex vector representing the received signal of the *i*th radar. The convolution operation in Equation (3) can be replaced with matrix multiplication by defining the convolution matrix:

(4) $$G_{\theta_{i},H_{j}}=\left[\begin{array}{cccc} g_{\theta_{i},H_{j}}\left(1\right) & 0 & \cdots & 0\\ g_{\theta_{i},H_{j}}\left(2\right) & g_{\theta_{i},H_{j}}\left(1\right) & \ddots & \vdots \\ \vdots & g_{\theta_{i},H_{j}}\left(2\right) & \ddots & \vdots \\ g_{\theta_{i},H_{j}}\left(L_{g}\right) & \vdots & \ddots & 0\\ 0 & g_{\theta_{i},H_{j}}\left(L_{g}\right) & \ddots & g_{\theta_{i},H_{j}}\left(1\right)\\ \vdots & 0 & \ddots & g_{\theta_{i},H_{j}}\left(2\right)\\ \vdots & \vdots & \ddots & \vdots \\ 0 & 0 & 0 & g_{\theta_{i},H_{j}}\left(L_{g}\right) \end{array}\right]$$

where **G***_θ_i_,H_j__* is an *L_s_* × *L_u_* complex matrix. Equation (3) can therefore be written as:

(5) $$s_{i}=\alpha_{i}G_{\theta_{i},H_{j}}u_{i}+n_{i}$$

The transmit waveform **u***_i_* is restricted by the total energy constraint, which is given by:

(6) $$u_{i}^{H}u_{i}=E_{i}$$

where *E_i_* is the normalized transmit energy of the *i*th radar.

## 3. *GLR* Based Sequential Hypothesis Testing

One of the three major characteristics of cognitive radar is the preservation of the information content of radar returns [9]. Haykin [9] suggests that this can be realized using a Bayesian approach. Based on this idea, Goodman [10] proposed the integration of waveform design techniques [8] with a SHT framework [11]. The test is based on sequential observations and updates running in a closed-loop. It updates the probabilistic understanding of all the hypotheses after each illumination and then makes a decision on the next transmit signal. In this section, we extend the SHT framework to the case of centralized cognitive radar networks. In addition, since we lack knowledge of the parameter *α_i_*, a GLR based SHT framework is used instead of the one based on likelihood ratio.

### 3.1. Amplitude Factor and GLR

In our signal model Equation (5), the variance of additive noise can be measured offline for each radar, but we still lack the knowledge of the parameter *α_i_*. The likelihood ratio test cannot be applied to this problem. Instead, we use the GLR test in which the unknown parameter *α_i_* is replaced with its MLE. Although the GLR test is not optimal, it appears to work quite well in practice [22].

When the aspect angle *θ_i_* is known for Equation (5), the MLE of parameter *α_i_* under *H_j_* is [23]:

(7) $${\hat{\alpha }}_{i|H_{j}}={\left[{\left(G_{\theta_{i},H_{j}}u_{i}\right)}^{H}G_{\theta_{i},H_{j}}u_{i}\right]}^{-1}{\left(G_{\theta_{i},H_{j}}u_{i}\right)}^{H}s_{i}$$

and the generalized likelihood function under *H_j_* is given by:

(8) $$p_{i}(s_{i};{\hat{\alpha }}_{i|H_{j}},\theta_{i}|H_{j})=\frac{1}{{\left(\pi \sigma_{i}^{2}\right)}^{L_{s}}}\text{exp}[-\frac{1}{\sigma_{i}^{2}}{\left(s_{i}-{\hat{\alpha }}_{i|H_{j}}G_{\theta_{i},H_{j}}u_{i}\right)}^{H}\left(s_{i}-{\hat{\alpha }}_{i|H_{j}}G_{\theta_{i},H_{j}}u_{i}\right)]$$

The likelihood function is used to update the probability of each target hypothesis until a decision has been made. In practice, we use the estimated *θ̂_i_* from Equation (1) to replace the unknown *θ_i_*. Since the scatterer distribution changes slowly with respect to the aspect angle (the position and intensity of scatterers remain approximately unchanged within ten degrees [24]), our approximation is reasonable. This also gives us an opportunity to reduce the number of target templates from different aspect angles stored in the knowledge base. A database with the template from every three degrees is sufficient for practical applications [25]. By replacing the unknown *θ_i_* with the estimated *θ̂_i_*, the generalized likelihood function is then given by:

(9) $$p_{i}\left(s_{i};{\hat{\alpha }}_{i|H_{j}},{\hat{\theta }}_{i}|H_{j}\right)=\frac{1}{{\left(\pi \sigma_{i}^{2}\right)}^{L_{s}}}\text{exp}\left[-\frac{1}{\sigma_{i}^{2}}{\left(s_{i}-{\hat{\alpha }}_{i|H_{j}}G_{{\hat{\theta }}_{i},H_{j}}u_{i}\right)}^{H}(s_{i}-{\hat{\alpha }}_{i|H_{j}}G_{{\hat{\theta }}_{i},H_{j}}u_{i})\right]$$

### 3.2. Centralized Bayesian Updates and Sequential Test

Since no interference exists among the radars in the network, the joint likelihood function under *H_j_* is the product of all the likelihood functions in (9) as given by:

(10) $$p(s_{1},\cdots ,s_{N}|H_{j})=\prod_{i=1}^{N}p_{i}\left(s_{i};{\hat{\alpha }}_{i|H_{j}},{\hat{\theta }}_{i}|H_{j}\right)$$

where *N* is the total number of radars in the network or the number of radars covering the target position if the detection area is not completely covered by the network. After every radar has performed an observation, the likelihood functions are gathered to update the probability of each target hypothesis *H_j_*. If we let *P*^(*k*−1)^(*H_j_*) represent the probability for *H_j_* before the *k*th observation, the posterior probability after executing the *k*th observation is given by:

(11) $$P^{\left(k\right)}(H_{j})=\frac{P^{\left(k-1\right)}(H_{j})\prod_{i=1}^{N}p_{i}(s_{i}^{\left(k\right)};{\hat{\alpha }}_{i|H_{j}}^{\left(k\right)},{\hat{\theta }}_{i}^{\left(k\right)}|H_{j})}{\sum_{j=1}^{M}[P^{\left(k-1\right)}(H_{j})\prod_{i=1}^{N}p_{i}(s_{i}^{\left(k\right)};{\hat{\alpha }}_{i|H_{j}}^{\left(k\right)},{\hat{\theta }}_{i}^{\left(k\right)}|H_{j})]}$$

The radar network continuously interrogates the target channel and updates the probability of each hypothesis until the time when hard decisions can be made with adequate confidence. Let *γ_m,n_* for *m* ≠ *n* be the desired probability of incorrectly selecting *H_n_* given that *H_m_* is true [12]. The GLR between *H_m_* and *H_n_* can be calculated as:

(12) $$\Lambda_{m,n}^{\left(k\right)}=\frac{P^{\left(k\right)}(H_{m})}{P^{\left(k\right)}(H_{n})}$$

The experiment terminates and *H_j_* is selected to be true when the condition:

(13) $$\Lambda_{j,n}^{\left(k\right)}>\frac{1-\gamma_{j,n}}{\gamma_{j,n}}\;\text{for all}\;n\neq j$$

is met for some *j* [10]. If the condition is not met for any of the hypotheses, another illumination cycle commences.

## 4. Adaptive Waveform Design

Another major characteristic of cognitive radar is the feedback structure from the receiver to the transmitter [9]. Based on the prior probability of each hypothesis obtained from previous tests, the transmit waveform can be optimized to enhance system performance and efficiency. In this section, we provide two adaptive waveform design techniques for extended target recognition. One is the eigen-waveform solution proposed by Guerci [8] and Goodman [10]. The waveform is designed to maximize the weighted average Euclidean distance between all the target hypotheses where the probabilities of the hypotheses are used as the weighting coefficients. This works quite well in situations where the amplitude of the ideal echo is known a priori. However, the method is not suitable for situations with amplitude fluctuation where the parameter *α_i_* is unknown. To solve this problem, we propose a minimum correlation algorithm for waveform design.

### 4.1. Eigen-waveform Solution

In situations with no amplitude fluctuation (*α_i_* = 1), a provably optimal transmit waveform for the *M* = 2 case is derived by Guerci [8]. The transmit signal should maximize the Euclidean distance between the mean values of the likelihood functions given by:

(14) $$d^{2}=u_{i}^{H}{\left(G_{{\tilde{\theta }}_{i},H_{1}}-G_{{\tilde{\theta }}_{i},H_{2}}\right)}^{H}\left(G_{{\tilde{\theta }}_{i},H_{1}}-G_{{\tilde{\theta }}_{i},H_{2}}\right)u_{i}$$

The unknown parameter *α_i_* is set to 1 in Equation (14), which indicates that the amplitude of the ideal echo is known a priori. The predicted target aspect angle with respect to the *i*th radar at the time of upcoming transmission is denoted by *θ̃_i_*. Prediction is required when the target is moving at high speed. Otherwise, it can be replaced with the latest estimated *θ̂_i_*. The optimal waveform under energy constraint Equation (6) which maximizes Equation (14) is the eigenvector corresponding to the maximum eigenvalue of the target autocorrelation matrix defined as:

(15) $$\Omega_{i}={\left(G_{{\tilde{\theta }}_{i},H_{1}}-G_{{\tilde{\theta }}_{i},H_{2}}\right)}^{H}\left(G_{{\tilde{\theta }}_{i},H_{1}}-G_{{\tilde{\theta }}_{i},H_{2}}\right)$$

When *M* > 2, the autocorrelation matrix is suggested to be in the form:

(16) $$\Omega_{i}=\sum_{m=1}^{M-1}\sum_{n=m+1}^{M}\omega_{m,n}{\left(G_{{\tilde{\theta }}_{i},H_{m}}-G_{{\tilde{\theta }}_{i},H_{n}}\right)}^{H}\left(G_{{\tilde{\theta }}_{i},H_{m}}-G_{{\tilde{\theta }}_{i},H_{n}}\right)$$

where *ω_m,n_* is a weighting factor [8]. The transmit waveform is designed to maximize the weighted average Euclidean distance of each binary pair, which is the eigenvector corresponding to the maximum eigenvalue of matrix **Ω***_i_*. Using the target probabilities, Goodman [10] compared two weighting factor *ω_m,n_* options, *ω_m,n_* = *P_m_* + *P_n_* and *ω_m,n_* = *P_m_P_n_*, and found that the second weighting coefficient provides better performance. However, since we have no idea about the value of unknown parameter α*_i_*, it should be set to 1 if the eigen-waveform solution is applied directly.

### 4.2. Minimum Correlation Algorithm

**Figure 3.**
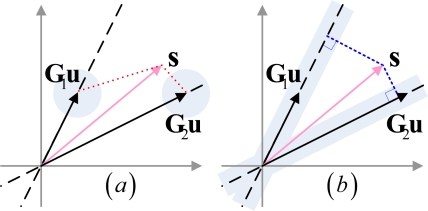
Multidimensional space for received signal under two target hypotheses. The situation without amplitude fluctuation is shown in **(a)** and the situation with amplitude fluctuation is shown in **(b)**.

To solve the problem of amplitude sensitivity, we suggest using the minimum correlation criterion instead of the maximum Euclidean distance. Figure 3 shows the multidimensional space for the received signal under two target hypotheses, where Figure 3(a) represents the situation without amplitude fluctuation and Figure 3(b) represents the one with amplitude fluctuation.

As shown in Figure 3(a), since the ideal echo signals **G**_1_**u** and **G**_2_**u** are exactly known, the likelihood ratio between the two hypotheses is determined by the Euclidean distance between **s** and **G**_1_**u** and the Euclidean distance between **s** and **G**_2_**u**. This leads to the idea of designing the transmit waveform to maximize the weighted average Euclidean distance. However, in situations with amplitude fluctuation, shown in Figure 3(b), the GLR between the two hypotheses is determined by the perpendicular distance from **s** to the axis of **G**_1_**u** and the axis of **G**_2_**u**. The transmit waveform should be designed to make the axis of **G**_1_**u** and **G**_2_**u** perpendicular to each other, or, in other words, to minimize the correlation between **G**_1_**u** and **G**_2_**u**. When *M* > 2, according to the eigen-waveform solution, the transmit waveform **u***_i_* is designed to minimize the weighted average of correlation between each binary pair. Thus, in our optimization approach we aim to achieve:

(17) $$u_{i}=\text{arg}\;\underset{u}{\text{min}}\sum_{m=1}^{M-1}\sum_{n=m+1}^{M}P_{m}P_{n}\left|u^{H}G_{{\tilde{\theta }}_{i},H_{m}}^{H}G_{{\tilde{\theta }}_{i},H_{n}}u\right|$$

subject to the constraint in (6), where *P_m_* and *P_n_* are the posterior probabilities of target hypothesis *H_m_* and *H_n_*.

## 5. Results

In this section, we demonstrate the benefits of a cognitive radar network for extended target recognition by comparing it to one without a feedback structure. We also compare the performance of the different adaptive waveform design approaches described in Section 4. To evaluate the performance of the closed-loop system, 500 different sets of targets are generated. Each set includes *M* = 4 target hypotheses. For each hypothesis, a two-dimensional target with multiple reflection centers is randomly generated according to:

(18) $$g_{H_{j}}\left(\tau_{x},\tau_{y}\right)=\sum_{l=1}^{L}\rho_{H_{j},l}\delta \left(\tau_{x}-\tau_{x,H_{j},l}\right)\delta \left(\tau_{y}-\tau_{y,H_{j},l}\right)$$

where the number of reflection centers *L* = 5, the reflection coefficients *ρ_H_j_,l_* are the samples of a zero-mean complex Gaussian distribution with unit variance, *δ*() denotes the Dirac delta function, and the locations of the reflection centers are the samples of uniform distribution in a circular region. The diameter of the circle equals the length of the target impulse response *L_g_*. From every aspect angle, the impulse response represents the projection of the reflection centers onto the radar LOS [26], which is given by:

(19) $$g_{\theta_{i},H_{j}}\left(t\right)=\sum_{l=1}^{L}\rho_{H_{j},l}\delta \left(t-\tau_{x,H_{j},l}\;\text{cos}\;\theta_{i}-\tau_{y,H_{j},l}\;\text{sin}\;\theta_{i}\right)$$

Since the Dirac delta function in Equation (19) is not practical and cannot be sampled in discrete time, the continuous impulse response is filtered using an ideal low-pass filter with bandwidth *B* = 1 and sampled with an interval of *T_s_* = 1. The elements of the impulse response vector **g***_θ_i_,H_j__* are given by:

(20) $$g_{\theta_{i},H_{j}}\left(n\right)=\sum_{l=1}^{L}\rho_{H_{j},l}\text{sinc}\left(n-(L_{g}+1)/2-\tau_{x,H_{j},l}\;\text{cos}\;\theta_{i}-\tau_{y,H_{j},l}\;\text{sin}\;\theta_{i}\right)$$

where *n* = 1,2 ⋯ *L_g_* and sinc() denotes the normalized sinc function. The impulse response vector is then normalized to unit energy. The specified error rate in SHT is *γ_m,n_* = 0.01 for all hypotheses and the prior probability *P*^(0)^ (*H_j_*) is set to 1/*M* for every *j*. The length of all impulse responses and waveform vectors is *L_u_* = *L_g_* = 31. In the observation process, the additive noise **n***_i_* is randomly generated with variance $\sigma_{i}^{2}=1$ and the amplitude factor *α_i_* is the sample of a zero-mean complex Gaussian distribution with unit variance.

### 5.1. Adaptive Waveform Design

**Figure 4.**
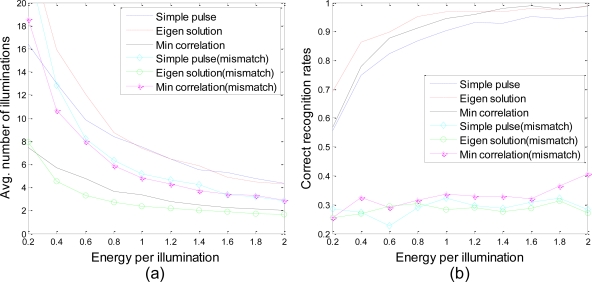
**(a)** Average number of illuminations to reach a decision and **(b)** correct recognition rates at the time when a decision has been made *vs.* energy per illumination for different waveform design approaches.

For Figure 4, three radars (located at *R⃗*_1_ = 0*x̂* + 0*ŷ*, *R⃗*_2_ = 10*x̂* + 0*ŷ*, and *R⃗*_3_ = 0*x̂* + 10*ŷ* km) form the network. The target is located at *R⃗* = 10*x̂* + 10*ŷ* km and is moving with velocity *v⃗* = 100*x̂* + 0*ŷ* m/s. The position and velocity of the target are assumed to be exactly known by the radars.

Figure 4(a) shows the average number of iterations required for each waveform design approach to reach a decision as a function of transmit energy while Figure 4(b) shows the correct recognition rates of the decisions. Since GLR is used in the Bayesian formulation instead of the likelihood function, the correct rates for different methods are no longer the same when the same desired incorrect probabilities are set. For each target set, two waveforms, a simple pulse and an eigen waveform, are used for comparison with the waveforms obtained by the proposed minimum correlation algorithm. A simple pulse is defined as $u=\sqrt{E/L_{u}}{\left[1,1,\cdots ,1\right]}^{T}$ which is a constant in the transmit duration. It does not change according to knowledge acquired from the environment, which represents a non-cognitive radar system. The eigen-waveform solution adaptively changes the transmit waveform to maximize the weighted average Euclidean distance between each hypothesis pair, where the weighting factor is the product of prior probabilities. In our algorithm, the waveform is designed to minimize the weighed correlation between the hypotheses. Both the eigen-waveform solution and the minimum correlation algorithm update the system’s understanding of the target after each observation and then optimize the waveform to match that understanding. To show the importance of using the GLR test in situations with amplitude fluctuation, the three waveform design techniques are also involved in a statistical model mismatch. With the same observations, the generalized likelihood function Equation (9) is replaced with the likelihood function that assumes the amplitude factor *α_i_* is known to be 1, which is not true.

As shown in Figure 4(b), for all three methods without considering actual amplitude fluctuations (mismatch), the correct recognition rates are approximately equal to 0.25 (1/*M*), which is the same as the probability of blind random selection. From Figure 4, it is clearly demonstrated that both of the closed-loop waveform design methods perform better than the approach transmitting a simple pulse. With approximately the same number of illuminations, the eigen-waveform solution achieves much higher correct rates. Meanwhile, the minimum correlation method not only reduces the average number of illuminations but also enhances recognition accuracy. Nevertheless, it is difficult to make a judgment between the two. Compared with the eigen-waveform solution, our method requires fewer illuminations, but performs higher probabilities of failure.

**Figure 5.**
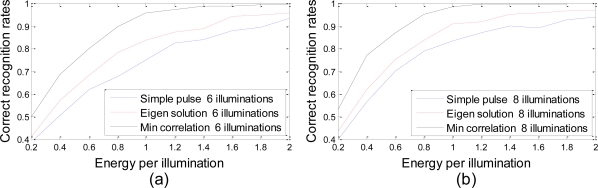
Correct recognition rates after six **(a)** or eight **(b)** illuminations *vs.* energy per illumination. The hypothesis with maximum probability is selected to be true after six **(a)** or eight **(b)** illuminations.

To make a fair judgment, an additional experiment is performed, in which all three methods execute the same number of illuminations. No hard decision is made by the SHT framework. The probability of each hypothesis is updated repeatedly after each illumination using the Bayesian formulation. Once the number of illuminations reaches the maximum, the hypothesis with the greatest probability is selected to be true. Figure 5 presents the correct recognition rates of 500 different sets after six illuminations and eight illuminations. Our method shows the highest recognition accuracy among the three while the approach transmitting a simple pulse shows the lowest. In addition, for each waveform design approach, the greater the number of illuminations is, the higher the correct recognition rates become.

**Figure 6.**
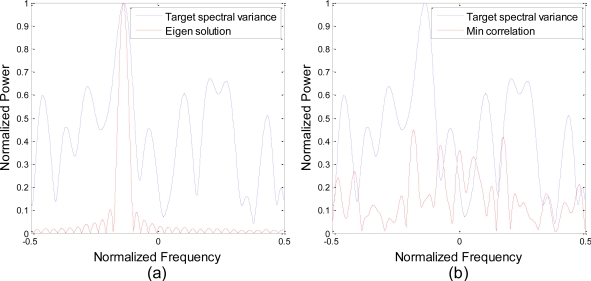
Waveform spectra compared to target spectral variance. Eigen-waveform solution is shown in **(a)** and the minimum correlation algorithm is shown in **(b)**.

In Figure 6, the spectrum of both the waveforms designed by the eigen-waveform solution and the minimum correlation algorithm are compared with the weighted spectral differences between the four impulse responses. To maximize the weighted Euclidean distance, eigen waveform focuses most of its energy on the maximum response frequency, since the Fourier transform preserves the Euclidean distance between signal and its spectra. However, our minimum correlation algorithm seems to have no significant relation with the target spectral variance, since it aims to achieve minimum correlation between the echo signals.

**Figure 7.**
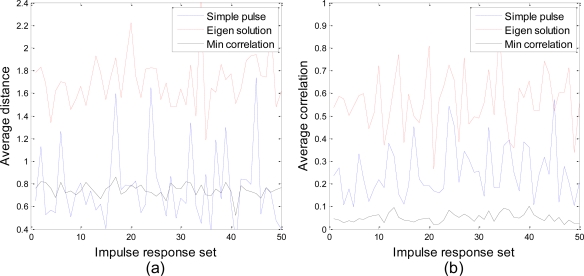
Comparison of **(a)** the average Euclidean distance and **(b)** the average correlation between the ideal echoes for different waveform design techniques.

The average Euclidean distance and the average correlation between the ideal echoes generated by different methods are shown in Figure 7, where 50 target impulse response sets are randomly generated according to previous descriptions as test samples.

As seen in Figure 7, the eigen waveforms clearly produce echoes with largest average distance among all the transmit waveforms. However, the eigen solution also causes highest correlation in the echo set. With the presence of unknown parameter *α_i_* described in our signal model (5), echoes with high correlation can hardly be distinguished from each other, since the amplitude and the initial phase of the signal no longer contain any information. The echoes of simple pulse show a volatile average distance since no adaptation is performed. A more stable average distance is acquired by our minimum correlation algorithm because the transmit energy is widely distributed in the passband. Despite the fact that the echoes of our algorithm show lower average distance than that of the eigen solution, our algorithm outperforms any other method in the comparison of average correlation, which leads to better recognition performance in the situations with amplitude fluctuation.

**Figure 8.**
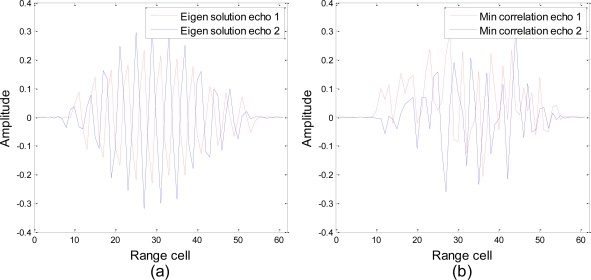
Ideal echoes generated by **(a)** the eigen-waveform solution and **(b)** the minimum correlation algorithm in a scenario with only two target hypotheses.

The ideal echoes in a scenario with only two target hypotheses are presented in Figure 8 to show the characteristics of different waveform design techniques in a more intuitive way. As we can see, the echoes corresponding to the eigen solution have more energy than the echoes corresponding to the minimum correlation algorithm, and the distance between the eigen solution echoes is also much larger than the distance between the minimum correlation echoes, which is consistent with the results shown in Figure 7(a). However, significant correlation is found between the eigen waveform echo 1 and the eigen waveform echo 2. It is very difficult to distinguish the two signals, if the eigen solution echo 1 is multiplied by a factor “−1”. The echo signals have a tendency to be opposite to each other about the origin in the multidimensional space, since the eigen solution aims to maximize the Euclidean distance between the two signals.

### 5.2. Estimation Variance of Target Aspect Angle

In previous experiments, the position and velocity of the target are assumed to be known by the radars, which means that the target aspect angle for the *i*th radar in Equation (1) is known. To show the influence of the estimation variance of aspect angle on system performance, the direction of the estimated target velocity $\hat{\vec{v}}$ is assumed to be the sample of a uniform distribution on [arg(*v⃗*) − Δ*θ*/2, arg(*v⃗*) + Δ*θ*/2], where Δ*θ* is the interval of the uniform distribution.

**Figure 9.**
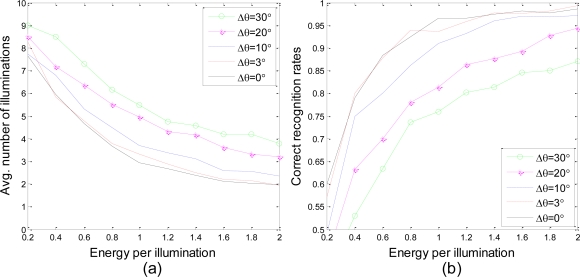
**(a)** Average number of illuminations to reach a decision and **(b)** correct recognition rates at the time when a decision has been made *vs.* energy per illumination for different estimation variances of the target aspect angle.

In Figure 9, the proposed waveform design approach is tested with 500 target sets in situations with different Δ*θ*, while the other conditions are the same as those from the previous experiments. As shown in this figure, higher correct recognition rates are achieved with fewer average illuminations in the situation of higher target aspect angle accuracy. If we assume that the variance of the measured Doppler velocity is the same for each radar, higher accuracy in target velocity can be acquired by increasing the number of radars in the network, which will lead to better system performance. It is also clear that the average number of illuminations and the correct recognition rates for both Δ*θ* = 0° and Δ*θ* = 3° are very close to each other. The feasibility of building a knowledge base with target templates every three degrees is proved once again.

### 5.3. Number of Radars

**Figure 10.**
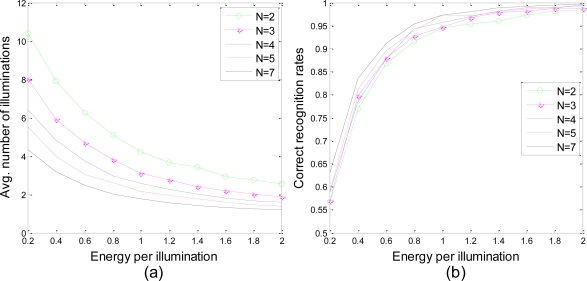
**(a)** Average number of illuminations to reach a decision and **(b)** correct recognition rates at the time when a decision has been made *vs.* energy per illumination for different numbers of radars in the network.

Figure 10 shows the performance of the proposed waveform design algorithm applied to centralized cognitive radar networks with different numbers of radars. The target is still located at *R⃗* = 10*x̂* + 10*ŷ* km and is moving with velocity *v⃗* = 100*x̂* + 0*ŷ* m/s while the radars are located at *R⃗*_1_ = 0*x̂* + 0*ŷ*, *R⃗*_2_ = 10*x̂* + 0*ŷ*, *R⃗*_3_ = 0*x̂* + 10*ŷ*, *R⃗*_4_ = 20*x̂* + 0*ŷ*, *R⃗*_5_ = 0*x̂* + 20*ŷ*, *R⃗*_6_ = 20*x̂* + 10*ŷ*, and *R⃗*_7_ = 10*x̂* + 20*ŷ* km. The first *N* radars in the queue are selected to work while the others remain idle. We also assume that the position and the velocity of the target are exactly known. As shown in Figure 10(b), the recognition accuracies are approximately the same for different number of radars in the network. In Figure 10(a), the average number of illuminations apparently decreases monotonically with increasing numbers of radars in the network. However, the total energy transmitted from all the radars to reach a decision still increases.

## 6. Conclusions

We have extended the idea of integrating waveform design techniques with a SHT framework for target recognition [10] to the case of a centralized cognitive radar network. Several issues, including the target-aspect sensitivity and the echo amplitude fluctuation, have been considered and solved. The GLR was employed in the SHT framework to update the joint probabilities of target hypotheses because of the unknown amplitude factor. The performance of three waveform design approaches, a non-adaptive method transmitting a simple pulse, the eigen-waveform solution, and our minimum correlation algorithm, are compared using simulation. The advantage of both adaptive waveform design technologies based on the latest knowledge about the target was substantial, and our minimum correlation algorithm outperformed the eigen-waveform solution. Moreover, the influence of system parameters on recognition performance is shown by simulations using different estimation variances of target aspect angle and different number of radars.
